# Constructing a hospital post-stroke depression management protocol by studying the management of post-stroke depression in a hospital setting

**DOI:** 10.1192/j.eurpsy.2021.1825

**Published:** 2021-08-13

**Authors:** N. Ghoshal, A. Sett

**Affiliations:** Fairfield General Hospital, Northern Care Alliance, Bury, United Kingdom

**Keywords:** post stroke depression, management protocol

## Abstract

**Introduction:**

A stroke is a potentially debilitating event which can render the victim unable to perform many tasks and functions, significantly decreasing their quality of life. This, in addition to emotional/mental changes post-stroke, can lead to a phenomenon known as “post stroke depression” (PSD), characterised by persistent low mood following a stroke.

**Objectives:**

This study aims to amalgamate recommendations based on national guidelines and previous literature, in addition to an original inpatient study of stroke patients within a hospital, to construct a standardised protocol of the management of PSD in the hospital setting.

**Methods:**

248 patients who had been treated for stroke within a hospital were analysed using hospital notes to assess for incidence of PSD, in-hospital management, and outpatient follow-up. In addition a literature search was conducted and national guidelines were consulted to develop a PSD management protocol.

Figure 1: Post stroke depression management protocol.

**Results:**

While 8% (20/248) of stroke patients experienced low mood immediately post stroke, 45% (9/20) of these patients did not receive any therapy or drug treatment, 80% (16/20) did not receive any outpatient monitoring of their mood and 100% of patients received no outpatient monitoring of newly commenced antidepressants.

**Conclusions:**

Using the results and literature search, a PSD management protocol, encompassing both appropriate in-hospital therapy and robust outpatient monitoring, was developed (Figure 1). We hope that through this, hospital care of PSD can be improved and optimised, in order for victims of PSD to receive the best possible, evidence-based care available to treat this potentially devastating condition.

**Figure:**
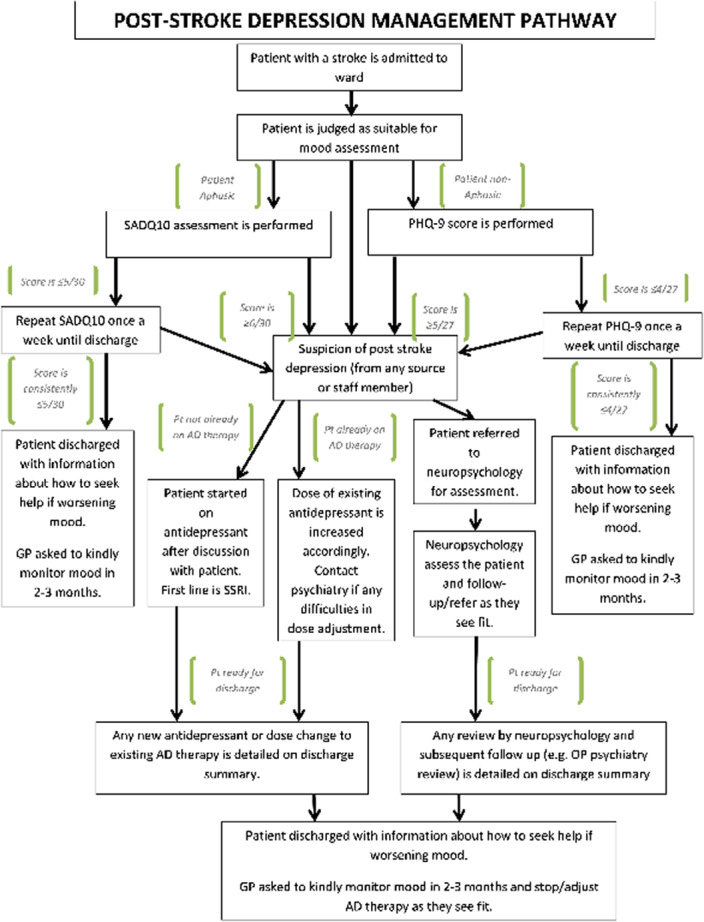

**Disclosure:**

No significant relationships.

